# HideRNAs protect against CRISPR-Cas9 re-cutting after successful single base-pair gene editing

**DOI:** 10.1038/s41598-022-13688-y

**Published:** 2022-06-10

**Authors:** Tim J. W. Harmsen, Colin E. J. Pritchard, Joey Riepsaame, Henri J. van de Vrugt, Ivo J. Huijbers, Hein te Riele

**Affiliations:** 1 Division of Tumor Biology and Immunology, The Netherlands Cancer Institute, Plesmanlaan 121, 1066 CX Amsterdam, The Netherlands; 2 Mouse Clinic for Cancer and Aging Research, The Netherlands Cancer Institute, Plesmanlaan 121, 1066 CX Amsterdam, The Netherlands; 3 Present Address: Plant Sciences and Natural Products, Institute of Biology Leiden (IBL), Leiden University, Sylviusweg 72, 2333 BE Leiden, The Netherlands; 4 Present Address: Sir William Dunn School of Pathology, University of Oxford, South Parks Road, OX1 3RE Oxford, UK; 5 Present Address: Department of Clinical Genetics, Section Oncogenetics, De Boelelaan 1118, 1081 HV Amsterdam, The Netherlands

**Keywords:** Genetic engineering, Targeted gene repair

## Abstract

Promiscuous activity of the *Streptococcus pyogenes* DNA nuclease CRISPR-Cas9 can result in destruction of a successfully modified sequence obtained by templated repair of a Cas9-induced DNA double-strand break. To avoid re-cutting, additional target-site-disruptions (TSDs) are often introduced on top of the desired base-pair alteration in order to suppress target recognition. These TSDs may lower the efficiency of introducing the intended mutation and can cause unexpected phenotypes. Alternatively, successfully edited sites can be protected against Cas9 re-cutting activity. This method exploits the finding that Cas9 complexed to trimmed guideRNAs can still tightly bind specific genomic sequences but lacks nuclease activity. We show here that the presence of a guideRNA *plus* a trimmed guideRNA that matches the successfully mutated sequence, which we call hideRNA, can enhance the recovery of precise single base-pair substitution events tenfold. The benefit of hideRNAs in generating a single point mutation was demonstrated in cell lines using plasmid-based delivery of CRISPR-Cas9 components and in mouse zygotes injected with Cas9/guideRNA plus Cas9/hideRNA ribonucleoprotein complexes. However, hRNA protection sometimes failed, which likely reflects an unfavorable affinity of hRNA/Cas9 *versus* gRNA/Cas9 for the DNA target site. HideRNAs can easily be implemented into current gene editing protocols and facilitate the recovery of single base-pair substitution. As such, hideRNAs are of great value in gene editing experiments demanding high accuracy.

## Introduction

Recent advances in nuclease-assisted gene modification technology are transforming fundamental and clinical science. Paramount to these developments are the unparalleled ease-of-use and high efficiency of engineered RNA-guided nucleases, chief among them *Streptococcus pyogenes* Cas9 (*sp*Cas9, Cas9)^1,2^. The location of a Cas9-induced DNA double-stranded break (DSB) is specified by a 20 nucleotide (nt) sequence (called: spacer) in the crRNA which together with the tracRNA produces the guideRNA (gRNA) that forms a ribonucleoprotein complex with Cas9. If this sequence matches a genomic sequence, the so-called protospacer, that is followed by an “NGG” ‘protospacer adjacent motif’ (PAM), a DSB is induced 3 base pairs (bp) upstream of the PAM^3,4^. Subsequent error-prone DSB repair can leave a scar that in many cases cripples the gene product. Alternatively, the DSB can be repaired by homology-directed repair (HDR) when a single- or double-stranded DNA template is provided. In this process, genetic information is transferred from the template to the genome, allowing pre-designed gene modification as subtle as the substitution of a single base pair.

Unfortunately, DSB induction has also been observed in sequences differing by 1 nt or more from the protospacer sequence and is at least partially due to a tolerance for spacer:protospacer mismatches^3,5–9^. The consequence of this “off-target” promiscuity is twofold: first, the induction of genomic DSBs may occur at sequences different from the original target site, potentially leading to unwanted gene disruptions; second, a single base-pair substitution introduced into the protospacer sequence by templated repair, may not necessarily render the modified sequence refractory to Cas9 activity. This “on target off-target activity” may cause re-cutting and destruction of the desired modified sequence, often frustrating the generation of single base-pair substitutions in cell lines and laboratory animals. To avoid re-cutting, additional mutations are often deliberately introduced in the repair template in order to disrupt the PAM or to increase the divergence with the gRNA spacer. However, these extra target-site-disrupting (TSD) mutations may not always be innocuous when they affect regulatory motifs, change codon usage or generate a non-synonymous codon. The latter is sometimes inevitable, e.g., when the PAM encodes a proline (see Fig. 6A, Mlh1 p.D304V substitution). Furthermore, not all PAM mutations preclude cutting, in particular when using Cas proteins with relaxed PAM requirements.

Promiscuous Cas9 activity, both at distant sites or on target, is undesirable and poses a major hurdle to the clinical maturation of Cas9 technology. Several strategies have been demonstrated to reduce off-target activity, such as engineering a requirement for the targeting of two guideRNAs for DSB induction (‘dual nickase’^9^ or FokI^10^ fusions), truncating the guide RNA^11^ or engineering the Cas9 protein^12–15^. Even though these implementations were successful in reducing overall off-target activity, break induction at some individual off-target sites remained^10,12,14,16^, or off-target activity at novel sites emerged^15,16^. Recently, single base-pair substitution with low level off-target events was accomplished by integrating Cas9-mediated single-strand break induction with reverse transcription of an RNA template^17^. Nonetheless, with ssODN-templated repair of Cas9-induced DSBs being the best studied and most versatile method of introducing base-pair substitutions, a requirement exists for techniques that can reduce off-target Cas9-activity.

In our attempts to generate single point mutations by templated DSB repair, we sought for a method to reduce the sensitivity of a successfully-modified locus to re-cutting by Cas9. Cas9 interaction with target DNA is strong and long lived^18^, and large portions of the target DNA including the PAM are buried in the gRNA/Cas9 ribonucleoprotein complex (RNP)^19^. This offers the intriguing possibility that a DNA target site can be ‘hidden’ by persistent binding to a non-active Cas9 RNP. One possibility is to use “dead Cas9” with mutated nuclease domains, which can bind a target site without inducing a DSB^20^. Alternatively, binding without cutting can also be achieved by wildtype Cas9 complexed with gRNAs containing a trimmed spacer^21–23^. Thus, Cas9 complexed to a gRNA with a trimmed spacer that perfectly matches the successfully mutated sequence, may confer protection against re-cutting by Cas9 bound to the full length gRNA with a now imperfectly matching spacer. In support of this idea, trimmed gRNAs have effectively been used to suppress off-target activity and also an example of on-target protection, increasing the recovery of a successful base-pair substitution, has been reported^24,25^. We here present an in-depth study of the performance of gRNAs with trimmed spacer sequences, which we will refer to as ‘hideRNAs’, to facilitate the recovery of single base-pair substitutions at different loci, both in cell lines and in murine zygotes.

## Results

### Re-cutting after successful base-pair substitution

**Figure 1:**
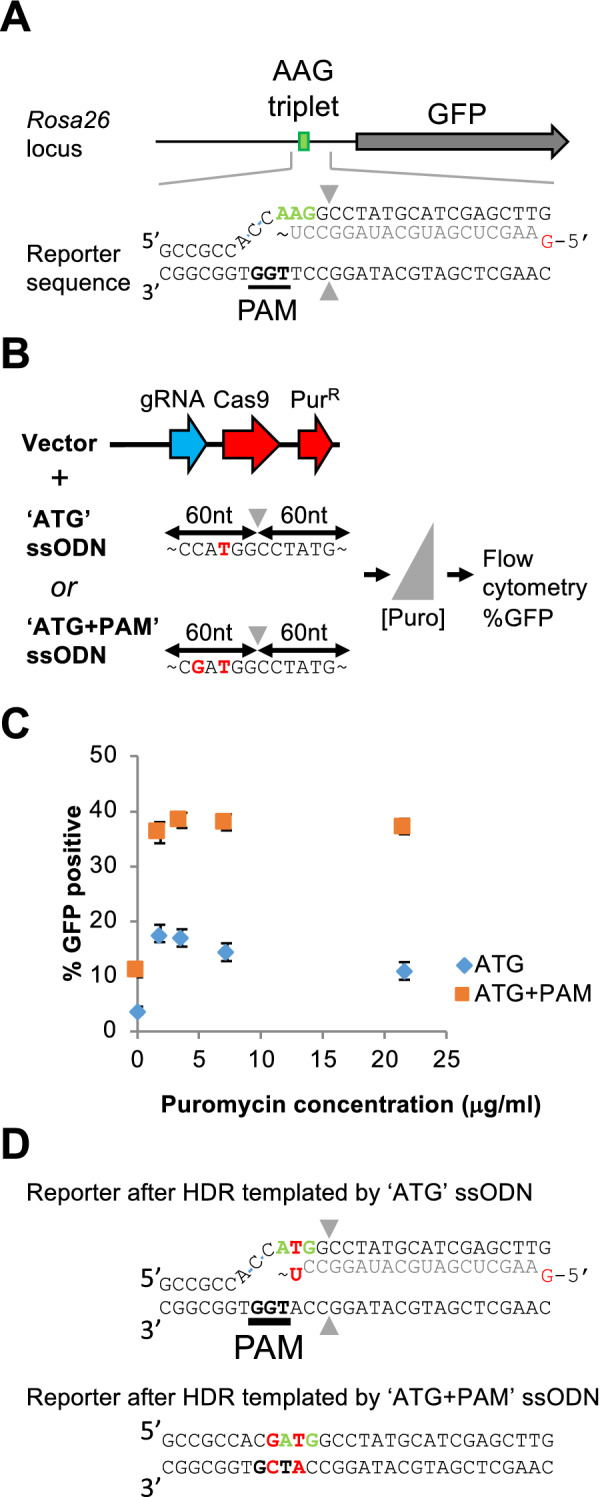
Re-cutting after CRISPR-Cas9-mediated single-nucleotide substitution. (**A**) In the *Rosa26* locus of mouse Embryonic Stem Cells, the enhanced GFP gene (grey arrow) is integrated, whose activity depends on conversion of an in-frame upstream AAG triplet (green rectangle) into ATG. Part of the reporter sequence is shown, with the AAG triplet marked in green. The bottom sequence shows the PAM (underlined); the upper stand is hybridized to the gRNA spacer sequence (light gray). The 5′ G (marked in red) of the RNA spacer does not hybridize to the target sequence. Gray triangles indicate the position of the double strand break. (**B**) Cas9 nuclease delivery system and ssODN repair templates. A puromycin acetyltransferase gene (encoding resistance to puromycin) was inserted into the Cas9/gRNA expression vector from Cong et al.^1^. Two 120 nt ssODN HDR templates were used: ‘ATG’ instructs conversion of the AAG into ATG; ‘ATG + PAM’ is identical to ‘ATG’ but also instructs disruption of the PAM. (**C**) Percentage of GFP positive cells determined by Flow cytometry as a function of the puromycin concentration used to select for transfected cells. Blue diamonds represent GFP percentages obtained with the ATG ssODN repair template; orange squares represent GFP percentages obtained with the ATG + PAM ssODN repair template. Error bars denote standard error of mean (n = 4). (**D**) Reconstruction of the target sequence after HDR-templated base-pair substitution. The top part shows that repair with ATG ssODN template still allows gRNA hybridization despite the single T-U mismatch. Repair with the ATG + PAM ssODN template induced PAM disruption, precluding Cas9-mediated re-cutting.

As a reporter for single base-pair substitution, we used mouse Embryonic Stem Cells (mESCs) carrying a single-copy sequence that encodes green fluorescent protein (GFP) but lacks a start codon. However, an in frame AAG triplet is present, which allows for GFP expression upon conversion into ATG (Fig. 1A)^26^.

To study single base-pair substitution by single-stranded oligodeoxyribonucleotide (ssODN)-templated homology-directed repair (HDR) of a DSB, we designed a single guide RNA (i.e., a fusion between crRNA and tracrRNA, here abbreviated as gRNA) that directs *Sp*Cas9 cleavage close to the AAG triplet (Fig. 1A). This gRNA was expressed from a vector that also encoded *Sp*Cas9 and puromycin acetyltransferase (the latter allowing enrichment of vector-transfected cells by puromycin selection) (Fig. 1B). We designed a 120 nt ssODN to template repair and direct the conversion of AAG to ATG. This conversion alters the protospacer by 1 bp (Fig. 1B). We transfected the vector and the ssODN repair template into the mESC reporter line, applying different concentrations of puromycin (Fig. 1B). Quantification of the fraction of GFP-positive cells by flow cytometry showed an increase in HDR efficiency with increased puromycin concentrations up to 3.6 μg/ml (Fig. 1C). However, HDR efficiency decreased when further increasing puromycin concentration.

Cas9 can induce DSBs at target sequences that differ slightly from the protospacer sequence, and this is stimulated by elevated Cas9 concentrations. In our system, high concentrations of puromycin may select for high Cas9 levels, which may promote re-cutting of the ATG containing sequence despite the imperfect match with the guideRNA (Fig. 1D). While successful conversion of AAG into ATG by HDR will activate GFP, subsequent re-cutting and NHEJ-mediated repair may disrupt this sequence and shift the ATG out of frame or remove it entirely, resulting in loss of GFP-expressing cells. To prevent this, the experiment was repeated with an ssODN template instructing an additional, PAM disrupting mutation (Fig. 1B). We now observed increased nucleotide substitution levels that did not decline at increased puromycin concentrations (Fig. 1C). This experiment demonstrates that a Cas9 target site of which the protospacer had been modified by HDR could still be cleaved by Cas9, and repaired in an error prone fashion. However, this can be prevented by the introduction of an additional Cas9 target site disrupting (TSD) mutation.

### HideRNAs

**Figure 2:**
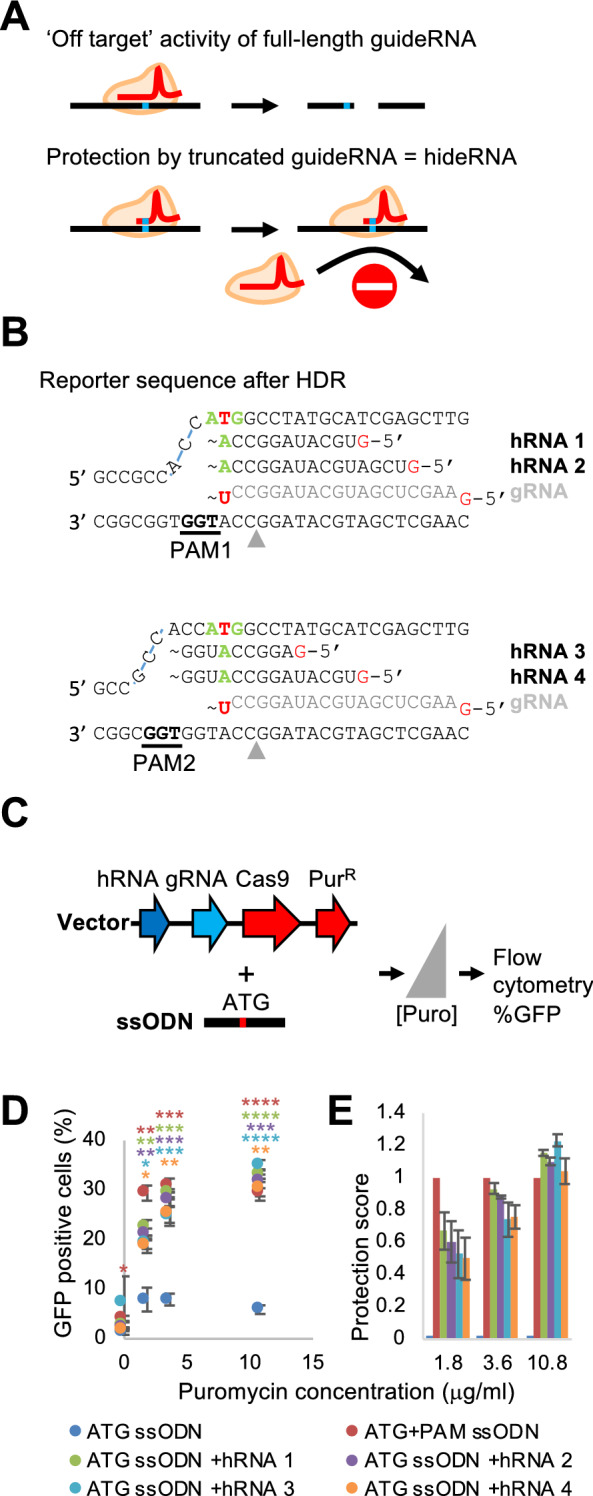
Protection against re-cutting by hRNAs. (**A**) Upper part: Cas9 in complex with a full-length gRNA (represented as red, curving line) can still induce a break despite the presence of a spacer-protospacer mismatch (blue rectangle). Lower part: a gRNA with truncated protospacer that has complete homology to the target sequence from the top part stably binds this sequence, but does not induce a break. It hides the target site from Cas9 bound to a full-length gRNA. (**B**) hRNAs with matching, truncated protospacer sequence protect the modified reporter sequence from re-cutting. hRNAs 1 and 2 use the original PAM (upper part); hRNAs 3 and 4 use an adjacent PAM. The start codon is marked green/red. Note that the sequence of the hRNA protospacers allows hybridization to the T of the ATG, (marked in green), whereas a T-U mismatch is formed with the protospacer of the gRNA (indicated in light grey). (**C**) Experimental setup for evaluating the re-cutting prevention capability of hRNAs. A vector expressing a hRNA and a gRNA is combined with an ATG-inducing ssODN. (**D**) GFP percentages obtained with 6 different conditions: gRNA + re-cutting-sensitive (ATG) or -protected (ATG + PAM) template; gRNA + hRNA 1–4 plus re-cutting sensitive (ATG) template. *p < 0.05, **p < 0.01, ***p < 0.001, ****p < 0.0001 (Welch one-sided test comparison with ‘ATG ssODN’ condition, color indicates for which condition this applies). Error bars denote standard error of mean (n = 3). (**E**) The protection score was calculated as follows: (G_i_ − G_sens_)/(G_PAM_ − G_sens_), with G_i_ the percentage of GFP positive cells for a sample, G_sens_ the percentage of GFP positive cells obtained with the gRNA and the re-cutting sensitive template, and G_PAM_ the percentage of GFP positive cells obtained with the gRNA and the re-cutting-protected template. Colors of each bar as in (**D**).

The introduction of a PAM mutation is highly effective in preventing re-cutting, but is often undesirable, e.g., when it causes a non-synonymous codon substitution. We therefore sought alternative ways to protect the successfully modified target site from promiscuous Cas9 activity. We hypothesized that a guideRNA with a truncated spacer sequence that perfectly matches the modified target sequence may promote tight binding of inactive Cas9 to the mutated sequence, thus conferring effective protection against re-cutting (Fig. 2A). To investigate if this strategy indeed prevents re-cutting and disruption of the repaired *GFP* reporter, we designed 4 truncated gRNAs that we call “hideRNAs” (hRNAs). The hRNAs consisted of truncated spacer sequences that were on the same strand as the original full-length gRNA, but included the AAG > ATG mutation (Fig. 2B). The protospacers of hRNAs 1 and 2 (length 12 and 16 nt), were associated with the same PAM as the gRNA (PAM1), whereas hRNAs 3 (10 nt) and 4 (15 nt) were associated with the adjacent PAM2 (Fig. 2B).

The four hRNAs were cloned separately into the vector from Fig. 1B adjacent to the gRNA expression cassette (Fig. 2C). Together with an HDR template instructing the ATG-creating nucleotide change but not the PAM-disrupting nucleotide change, we introduced these vectors into mESCs and varied Cas9/gRNA/hRNA levels by selecting with 3 different puromycin concentrations (Fig. 2C). We then compared the number of GFP-positive cells to that obtained with the experimental conditions from Fig. 1B (only gRNA + ATG or ATG + PAM ssODN). Strikingly, we observed that the presence of hRNAs strongly improved the recovery of GFP-positive cells with the ATG ssODN, reaching levels as high as obtained with the ATG + PAM ssODN (Fig. 2D). This effect was not caused by the utilization of plasmid-borne hideRNA encoding sequences as a template for HDR as omission of the ssODN did not yield GFP-positive cells above background (Supplementary Fig. 1). We calculated a “protection score” for each hRNA, 0 being no protection and 1 being as much protection as a PAM disrupting mutation (Fig. 2E). While we found protection scores around 0.5 for the different hRNAs at 1.8 μg/ml puromycin, protection was maximal (around 1) for the highest puromycin concentration tested. This demonstrates that the introduced ATG mutation could effectively be protected against re-cutting by any of the 4 hRNAs, obviating the requirement for an additional PAM-disrupting mutation.

### HideRNA parameters

**Figure 3:**
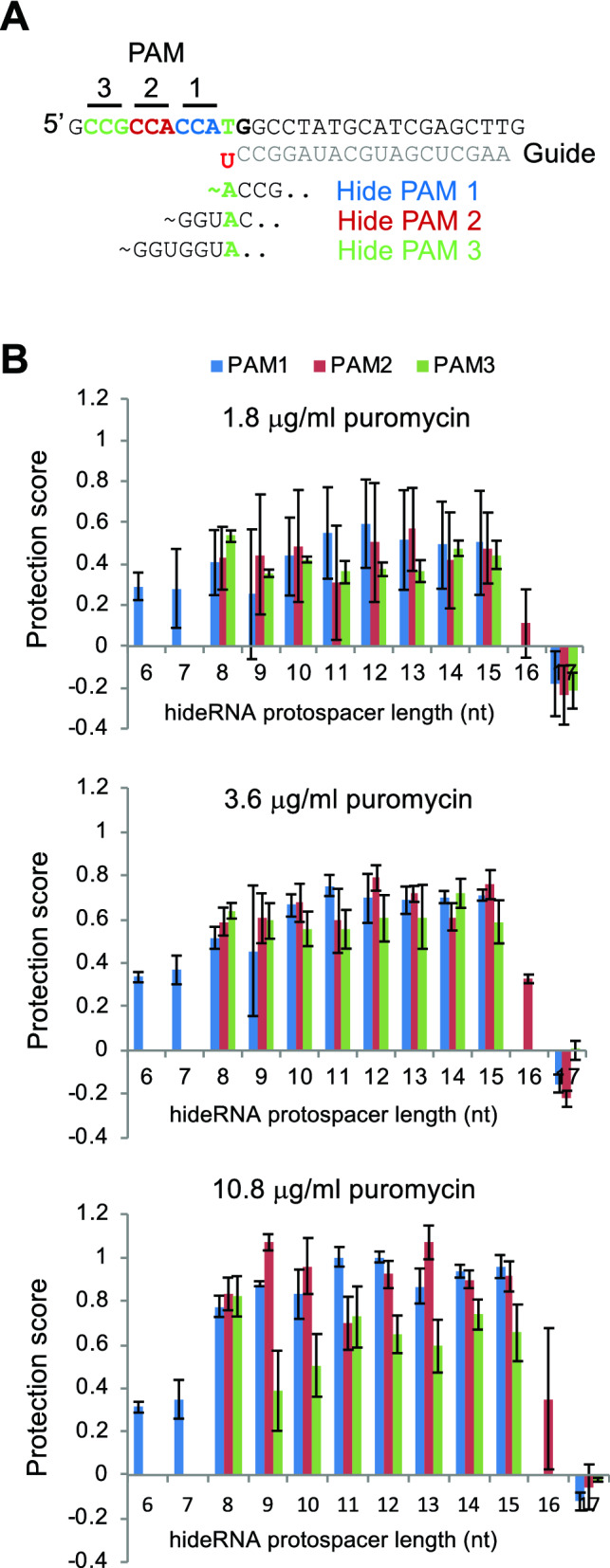
Parameters affecting hRNA performance. (**A**) Three hRNAs using different PAMs (1, 2 and 3, marked blue, red and green, respectively) and varying protospacer lengths were designed to protect the activated GFP reporter against re-cutting. Part of the GFP reporter sequence after repair with the ATG only template is shown (T of ATG marked green) and the PAMs. Part of the spacer sequence of the hRNAs that target PAMs 1–3 is shown, with the A that marks their specificity marked in green. (**B**) Protection scores of hRNAs 1–3 with different spacer lengths determined at three different puromycin concentrations. If the first nucleotide of the protospacer was not a G, a G was added to the sequence. The hRNA protospacer length does not take a mismatching 5′ G into account. Error bars denote standard error of mean (n = 3).

The four hRNAs tested seemed to perform equally well, despite being associated with two different PAMs and having different protospacer lengths. To identify parameters for best hRNA protection, we designed additional hRNAs for the PAM used by the original gRNA sequence, and two other PAMs on the same strand (Fig. 3A) and varied the length of the spacer. The protection scores (Fig. 3B) of these hRNAs were determined in the same way as in Fig. 2E. At intermediate puromycin concentration (3.6 μg/ml), most hRNAs had protection scores around 0.6. At the highest puromycin concentration (10.8 μg/ml), we observed protection scores approaching 1 for hRNAs associated with the two PAMs closest to the mutation. Spacer length seemed less critical (8–15 nucleotides were equally effective). The protection score of hRNAs associated with the PAM most distal from the mutation was not increased at this puromycin concentration. Interestingly, a sharp cut-off was seen for hRNAs with spacer lengths > 15 nt, showing no protection (16 nt) or even negative protection scores (17 nt). Possibly this reflects a DSB inducing capability of these hRNAs or increased binding to the non-modified sequence causing competition with the gRNA.

Besides hiding the modified target sequence, two non-specific mechanisms may contribute to reduced re-cutting. First, hideRNA-bound Cas9 is unavailable for binding gRNA. Thus, competition between hRNA and gRNA binding to Cas9 may reduce Cas9:gRNA re-cutting activity, irrespective of the protospacer sequence of the hRNA. To determine if this “sponge effect” existed, we tested the protection efficiency of 6 hRNAs with protospacer sequences unrelated to the original target site. Two hRNAs showed approximately 20% of the full protection obtained with a PAM-disrupting mutation, albeit at only one of the puromycin concentrations used (Supplementary Fig. 2). This indicated that occasionally competition for Cas9 binding between hRNA and gRNA may lower re-cutting incidence, although the contribution of this mechanism is modest. The second reason for decreased re-cutting activity may be related to cytotoxic or growth-retarding effects incited by the binding of large numbers of inactive Cas9 complexes to the DNA. Upon expression of some, but not all hRNAs, we noticed decreased numbers of living cells (Supplementary Fig. 3). If progressively higher cellular hRNA expression leads to decreasing cell viability, the surviving cell population may harbor a lower cellular concentration of catalytically active RNP with an according reduction in re-cutting propensity. However, we did not observe a correlation between protection efficiency and cell numbers, indicating this effect did not significantly contribute to hRNA protection.

These results indicate that apart from sequence specificity, further requirements for effective hRNAs are relaxed: different spacer lengths and different PAMs perform equally well against re-cutting. In many cases, only the PAM that is part of the original target site will be available and this offers good protection. Protection seems most efficient at high nuclease concentrations.

### Protection of a mutation outside the original target site

**Figure 4:**
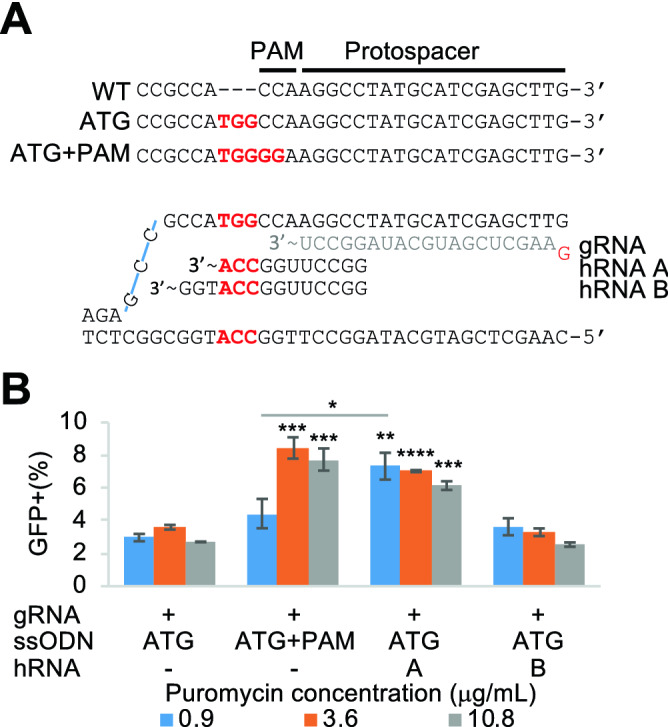
hRNAs protecting a mutation outside the target site. (**A**) Top: sequences of the reporter before (WT) and after ssODN-directed insertion of TGG (ATG) or TGG insertion plus PAM disruption (ATG + PAM). The ‘ATG’ template leaves the entire Cas9 target site intact and thus sensitive to re-cutting. Bottom: gRNA and hRNA A and B spacers are shown, interacting with the target sequence after TGG insertion. (**B**) HideRNAs can protect a mutation that is outside of the Cas9 target site. The percentages of GFP-expressing cells are scored after puromycin selection at 3 different concentrations for combinations of Cas9 and the gRNA, the ATG, ATG + PAM ssODN template and hRNA (**A**) or (**B**). Error bars denote standard error of mean (n = 3). *p < 0.05, **p < 0.01, ***p < 0.001, ****p < 0.0001 (Welch one-sided test, comparison with the ATG ssODN template).

We next investigated whether hRNAs could also protect mutations that do not modify the original Cas9 target site. We designed an ssODN repair template to insert a TGG triplet that creates an in-frame ATG upstream of the gRNA target site thus leaving the PAM and protospacer sequence intact (Fig. 4A). We designed a similar oligonucleotide template with additional PAM disrupting mutations (ATG + PAM). We hypothesized that HDR templated by the ‘ATG’ only ssODN would result in GFP expression, but subsequent re-cutting in the downstream unaltered Cas9 target site may result in end-joining-mediated disruption of GFP expression. Indeed, compared to the unprotected ATG template, the ‘ATG + PAM’ template increased the recovery of GFP-positive cells approximately twofold (Fig. 4B). We designed two hRNAs (A and B) using different PAMs (Fig. 4A) and examined if these were able to prevent re-cutting. Strikingly, we observed protection by hRNA A was as efficient as that obtained with the ATG + PAM template (Fig. 4B). hRNA B, which utilized a PAM that was 3 bp further away did not protect the modified allele from re-cutting. These results demonstrate that hRNAs can be utilized to protect mutations that are created outside of the Cas9 target site.

### HideRNA effectivity at endogenous genes

**Figure 5:**
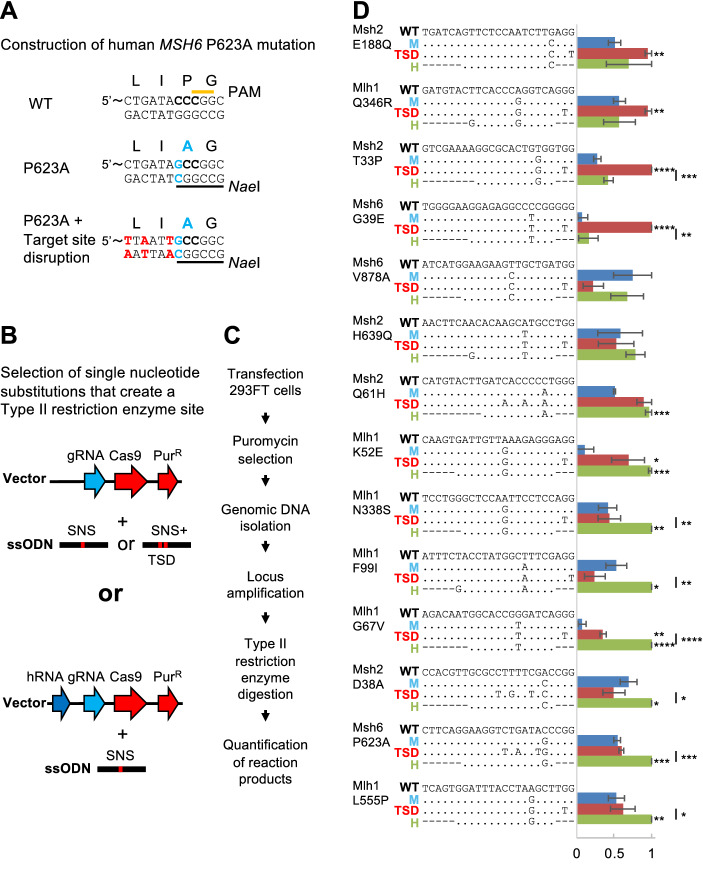
hRNA protection at endogenous loci. (**A**) Introduction of 1 nt substitution in the *MSH6* DNA MMR gene that results in a motif recognized by the Type II restriction endonuclease *Nae*I. Top: the wild-type sequence with single-letter amino acid code. The PAM is indicated with an orange line. Middle: one nucleotide change (in blue) changing the encoded amino acid from proline to alanine. In addition, an *Nae*I site is created (indicated with a black line). Bottom: additional, silent mutations have been instructed to disrupt the protospacer. Note that PAM disruption in this case would have disrupted the *Nae*I site. (**B**) Three different transfections were performed for each mutation: gRNA + template instructing a single nucleotide change (SNS), gRNA + template instructing SNS and additional Cas9 target site disrupting (TSD) mutations, or gRNA + hRNA + template instructing SNS. (**C**) Experimental workflow. Quantification of the reaction products was performed using the Caliper GX. (**D**) HideRNAs can be used to protect mutations generated at endogenous loci. On the left, the gene name and intended codon substitution is shown. For each mutation, 4 sequences are shown: the wildtype genomic CRISPR-Cas9 target sequence (WT), the sequence where the SNS has been introduced (marked with a blue ‘M’), the sequence with the SNS plus additional target site disruption (marked with red ‘TSD’), the hRNA spacer sequence. Dots denote sequence identity; dashes indicate that this sequence is not part of the hRNA protospacer. The scores are calculated based on the percentages of digested DNA. Amounts of cut and uncut DNA were quantified on the labchip caliper GX, and the fraction of DNA that contained a Type II restriction site was calculated as: (cut DNA/all DNA)*100%. The experiment was performed three times. Within each replicate, a ‘max-normalization’ was performed—fraction of DNA was divided by the value of the sample with the largest amount of digested DNA. Max normalizations for all replicates were averaged and shown with SEM. *p < 0.05, **p < 0.01, ***p < 0.001, ****p < 0.0001 (Welch one-sided test comparison with ‘M’ (no protection) condition, unless otherwise indicated).

We next assessed the generalizability of hRNAs for the protection of small mutations introduced with CRISPR-Cas9. We selected 15 naturally occurring variants in the human DNA MMR genes *Mut S homolog 2* and *6* (*MSH2*, *MSH6*) and *Mut L homolog 1* (*MLH1*) that can be generated by 1 nt changes that also create Type II restriction enzyme sites. For each mutation, guide RNAs were designed to introduce a DSB close to the intended mutation. We designed ‘unprotected templates’: 90 nucleotide long ssODN templates identical to the target sequence except for the desired single nucleotide substitution (SNS). We also designed templates with a target site disrupting mutation (TSD), which were identical to the SNS templates but included additional PAM or protospacer disrupting mutations (exemplified in Fig. 5A). These additional mutations did not disrupt the restriction enzyme site, and where within 5 bp of the DSB, as we previously found that longer distance could negatively affect the efficiency with which these additional mutations are introduced into the genome. TSDs were synonymous in 8 cases, non-synonymous in 5 cases and affecting an intronic sequence in 2 cases. In addition, we designed hRNAs for protection of each SNS. These hRNAs had the same PAM sequence as the gRNA and their protospacer was shortened to a 10–15 nucleotide sequence preceding the PAM and preferentially starting with a guanosine. If no natural guanosine was present, a 12 or 14 nucleotide sequence was selected and provided with a guanosine on the 5’ side. Cassettes expressing these hRNAs were cloned into their respective gRNA vectors to yield 14 vectors that expressed a gRNA and a hRNA. We then transfected 293FT cells with gRNA expressing vectors and ssODN templates with the intended SNS only or with an additional TSD. In addition, we transfected the 293FT cells with the gRNA- and hRNA-encoding vector and the SNS-only template (Fig. 5B). After transfection, we selected for uptake of the vector with 7.2 μl/ml puromycin. PCR fragments containing the target locus were incubated with the appropriate restriction enzyme, and the fraction of DNA that contained the modification was quantified (Fig. 5C). Digestion of the target locus, indicating HDR events had taken place ranged from 1 to 12% (the percentage of digested DNA of the total DNA). For each locus, we calculated a max-normalized HDR efficiency for the three different conditions, which is shown in Fig. 5D.

In only two cases (MSH6 V878A and MSH2 H639Q), the recovery of HDR events did not benefit from TSD or hRNA protection. The remaining cases benefited from TSD (6/14 cases) and/or from hRNA protection (8/14 cases). Somewhat remarkably, only 2 cases overlapped, i.e., benefited from both, TSD and hRNA protection (MLH1 K52E and G67V). Thus, in 4 cases (MSH2 E188Q, MLH1 Q346R, MSH2 T33P and MSH6 G39E) hRNAs did not reach the improvement achieved by TSD. Conversely, in 7 cases (MLH1 K52E, N338S, F99I, G67V and L555P, and MSH6 P623A, and MSH2 D38A) hRNAs offered the best protection, yielding 2–10fold improvement over the unprotected situation. Importantly, while in two cases target site disruption seemed to reduce efficiency with respect to the unprotected situation (MSH6 V878A: *p* = 0.07; MLH1 F99I; *p* = 0.11), the addition of a hRNA either gave the same efficiency (6/14 cases) or improved the efficiency 2–10fold (8/14 cases).

### HideRNAs increase the fraction of HDR in mouse zygotes

**Figure 6:**
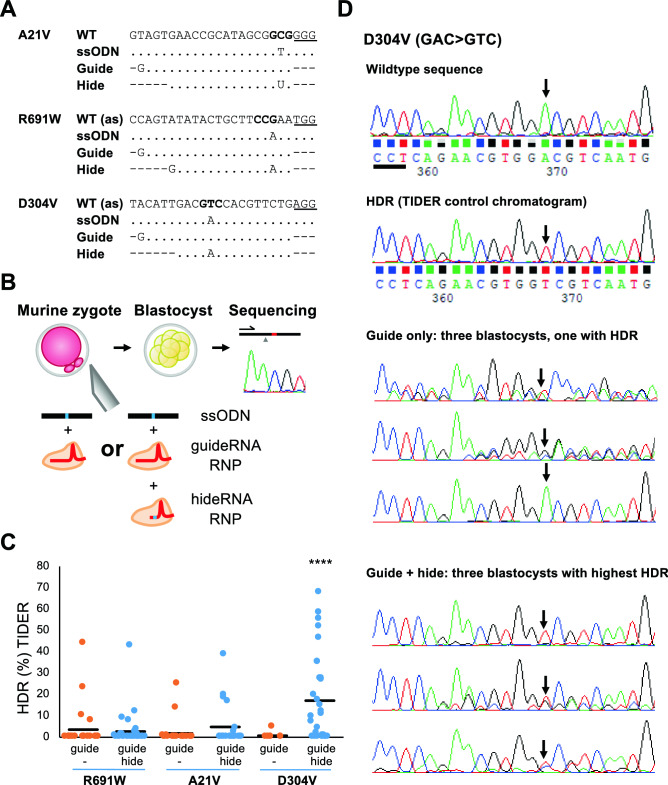
hideRNA protection in zygotes. (**A**) Sequences of wildtype (WT), oligonucleotide template (ssODN) and guide and hide RNAs for three different *Mlh1* mutations. (**B**) For each mutation two rounds of zygote injections were performed: ssODN plus RNP with guideRNA or ssODN plus RNP with guideRNA *and* RNP with hRNA. Injected zygotes were cultured to blastocyst stage and analyzed for the presence of the mutant allele by Sanger sequencing. (**C**) The percentage of HDR-deriving alleles was calculated per embryo by the TIDER algorithm. Horizontal bars indicate the average percentage of mutant allele. Number of blastocysts analyzed: R691W guide, n = 24; R691W guide + hide, n = 29; A21V guide, n = 12; A21V guide + hide, n = 21; D304V guide, n = 7; D304V guide + hide, n = 31. ****p < 0.00001 (Welch one-sided test). (**D**) Examples of the chromatograms produced by Sanger sequencing blastocyst gDNA from guide-only and guideRNA + hRNA injections to make the D304V mutation. The arrow indicates the position of the base-change achieved upon HDR with the oligonucleotide template and the black line indicates the PAM of the guide and hRNA (the orientation of the chromatogram sequence is complementary to the one depicted in **A**). With guide only, just one blastocyst showed HDR.

Subtle gene modification in mice can nowadays efficiently be achieved by injecting zygotes with Cas9 ribonucleoprotein complexes targeting the genomic DNA along with oligonucleotides templating the site-specific modification. Also here, TSDs are often added to improve the recovery of gene-edited animals, but this may have undesired effects. We therefore investigated whether hRNAs could be used to increase the frequency of ssODN-templated *single-nucleotide substitution* in murine zygotes. We designed guideRNAs, oligonucleotides and hRNAs to generate three different point mutations in the murine *Mlh1* gene, representing variants of uncertain significance found in suspected Lynch Syndrome patients (Fig. 6A). GuideRNA and hRNA Cas9 ribonucleoprotein (RNP) complexes were assembled in separate reactions. For every point mutation, we performed two rounds of mouse zygote injections: guideRNA RNPs with oligonucleotide and guideRNA *plus* hRNA RNPs with oligonucleotide (Fig. 6B). Injected zygotes were cultured until blastocyst stage and the fraction of HDR modification among the recovered alleles was determined by Sanger sequencing. It was apparent that the majority of chromatograms consisted of mixed sequences. To quantify the contribution of the sequence resulting from HDR, we algorithmically deconvoluted chromatograms with TIDER (Fig. 6C)^27^ and ICE (Supplementary Fig. 4A) (Hsiau et al*.*, bioRxiv 251082, accessed 20190609). Both algorithms determine fractions of wildtype, HDR-derived and indel-containing sequences. There was considerable overlap between the group of blastocysts that could be successfully deconvoluted by either algorithm (Supplementary Fig. 4B), but the number of deconvolutions with an R^2^ exceeding 0.8 was higher for the TIDER algorithm. The percentage of HDR alleles could be determined for 109 blastocysts out of 239 by both algorithms with an R^2^ of at least 0.8 for each individual chromatogram (Supplementary Fig. 4B), whereas the agreement between the two algorithms had an R^2^ of 0.91 (Supplementary Fig. 4C). However, in some cases, TIDER detected HDR alleles up to 20% that were not seen with ICE (Supplementary Fig. 4C).

The percentage of HDR alleles per blastocyst found by TIDER is shown in Fig. 6C, while a similar graph with ICE data, applying to fewer blastocysts, is shown in Supplementary Fig. 4A). In most cases the percentage of HDR alleles was lower than 50%, indicating that HDR events or re-cutting events disrupting HDR alleles took place at or beyond the 2-cell stage. Both, TIDER and ICE have a detection limit around 5%. When we only consider HDR percentages above 5%^27^ as reliably indicating successful base substitution, according to both, TIDER and ICE, the D304V substitution strongly benefited from the co-injection of a hRNA, with the presence of HDR alleles above 5% increasing from 0 (0 out of 7) to ± 45% of the blastocysts (Fig. 6C and Supplementary Fig. 4A). The chromatograms in Fig. 6D highlight the hideRNA effect on the level of individual blastocysts. The HDR mutation is only visible in one of the chromatograms of the three best-scoring blastocysts from the hideRNA-free condition (Guide only). Conversely, in the chromatograms of the three best-scoring blastocysts condition with hideRNA supplementation, the wildtype nucleotide is virtually absent (Guide plus hide). The benefit for R691W and A21V was modest, if present, with TIDER suggesting a slight benefit for A21V (2/22 to 4/21) (Fig. 6C) and ICE suggesting a slight benefit for R691W (1/19 to 5/29) (Supplementary Fig. 4A).

These results demonstrate that hRNAs not only can work with plasmid-based delivery of CRISPR-Cas9 components, but also with RNP complexes injected into zygotes, albeit not with 100% efficiency. The addition of a hRNA may thus be useful for the generation of transgenic mice carrying only a single base-pair substitution.

## Discussion

We explored and exploited a novel tool for repression of promiscuous Cas9 activity to increase the recovery of subtle base-pair substitutions introduced by ssODN-templated repair of a Cas9-induced DSB. Such minor substitutions, even when located in the original protospacer, do often not prevent targeting by the gRNA:Cas9 complex, which may lead to re-cutting and destruction of the modified allele. We reasoned that the modified allele may resist re-cutting when it is shielded by an inactive Cas9 RNP complex rendering it inaccessible to catalytically active Cas9 RNP. An inactive complex could be formed by Cas9 and a gRNA with a trimmed spacer that perfectly matches the modified site and therefore could bind with greater affinity than the mismatched gRNA:Cas9 complex. Using a disabled *GFP* gene as reporter for HDR-mediated gene editing, we found that four different trimmed gRNAs, which we dubbed hideRNA (or hRNA), expressed from a vector that also encoded Cas9 and the gRNA efficiently protected the intended single nucleotide substitution against re-cutting. A similar example of target site protection has been reported by Rose et al.^24^.

The vector system for delivering Cas9, the gRNA and the hRNA to the cell ensures a 100% co-transfection efficiency of the gRNA and the hRNA, but the amount of Cas9 protein is the same as that expressed with one guideRNA. Because of this, two additional processes in our experimental system could enact protection against re-cutting in a spacer-independent way^28^. Both, a “sponge effect” whereby hRNA binding lowers the concentration of catalytically active Cas9 complex and cytotoxicity due to the binding of large numbers of inactive Cas9 complexes to the DNA, had only a minor contribution, if any (Supplementary Figs. 2 and 3). Thus, the testing of random spacer sequences provides evidence that hRNAs exert their effect by physical protection of the modified target site through specific spacer-dependent binding. Consistently, in their off-target protection study, Rose et al. demonstrated that Cas9.dRNA (dead RNA, the equivalent of hRNA) complexes can directly shield off-target loci from Cas9.gRNA cleavage^24^.

This interpretation is in line with studies of Cas9’s structure and biochemistry addressing the mechanism that governs its interaction with DNA in the presence and absence of spacer:protospacer mismatches. Binding to a gRNA enables Cas9 to scan DNA for PAM sequences^18,29^. When a PAM is encountered, Cas9 locally melts the DNA, initiating spacer-protospacer hybridization close to the PAM^18,30^. If this hybridization is successful, the full length of the spacer is annealed, followed by nuclease domain activation and DSB induction^31–33^. Target binding has a higher tolerance for mismatches than cleavage^34,35^. Binding without break induction has been observed for gRNAs that have a protospacer shorter than 17 nt^21–23^. In vitro studies have demonstrated that the binding of Cas9 protects 24 bp of target DNA from DNAse I, including residues composing the PAM. These findings support a model in which the hRNA-bound Cas9 complex is able to bind DNA, but unable to activate the nuclease domains. This inactive complex hides the PAM residues from gRNA-bound Cas9, and so interferes with the first step of Cas9’s cleavage mechanism. Based on transcription stimulating ability as readout for Cas9 binding strength, hRNAs are expected to exhibit the same binding strength as guideRNAs on the same target DNA^22,23^. However, the original target sequence has the highest affinity for catalytically active Cas9/gRNA complex, while the modified sequence preferentially binds hRNA-bound inactive Cas9, which likely augments protection from re-cutting.

Investigating 14 single-nucleotide substitutions at three different genes in human cells revealed that hRNA-mediated protection was at least as efficient as TSD-mediated protection, but the two types of protection did not always overlap and in some cases, hRNA protection did not work. Importantly though, unlike TSDs, hRNAs never lowered the substitution efficiency. The exact parameters that govern efficient hRNA binding protection are at this moment unclear. By systematically investigating the efficiency of protection of a single mutation at a single target locus, we found that the length of the hRNA spacer is of little influence (protospacer lengths of 8–15 nucleotides were effective, Fig. 3), but the proximity of the hRNA PAM to the gRNA PAM may be important (Fig. 3). This suggests that hRNA protection efficacy depends on the relative DNA-binding kinetics of hRNA- and gRNA-complexed Cas9. This balance is probably sequence dependent and influenced by the specific spacer:protospacer mismatches resulting from successful gene editing. Nonetheless, we present a case where the hRNA strategy protected nucleotide changes introduced outside the original target site. In this case, protection was only conferred by a hRNA using a proximal PAM, but not by another hRNA that relied on a more distal PAM. Achieving protection in this situation may be more challenging as the hRNA faces stronger competition from the gRNA, which still has a perfect match with the target sequence. To improve the efficacy of hideRNA protection, the activity of the gRNA may be attenuated by deliberately introducing mismatches in its spacer sequence. Plasmon resonance studies addressing the DNA on- and off-rates of hRNA and gRNA RNPs may divulge rules for the most optimal hRNA design. Also, the increasingly better methods available for prediction of gRNA binding efficacy^36^ should be useful for predicting hRNA binding efficacy, so that hRNA protection efficiency in different gene editing situations may be optimized.

The vector delivery system ensures concomitant cellular uptake of hRNA and guideRNA, but can result in log-fold differences in cellular RNP concentrations as a consequence of low and high plasmid copy numbers, the latter being associated with increased off-target activity^5,6,28^. An alternative may be the delivery of CRISPR-Cas9 nucleases as an in vitro assembled RNP complex^37,38^. In fact, direct injection of RNP complexes and DNA templates into zygotes is currently the most popular route towards the generation of genetically-modified mice. We demonstrate here that also in this application the addition of hRNA-containing RNP can facilitate the recovery of genetically modified embryos in one of the three cases tested. Clearly further work is needed to optimize this application.

In addition to protecting templated mutations created by HDR, the hRNA concept may have more applications. It is becoming increasingly appreciated that template-free repair can sometimes produce desirable outcomes. However, these outcomes may be subject to re-cutting too, as protospacers with small indels compared to the spacer can still be targeted. We propose that hRNAs can be applied in these protocols to increase the frequency of a desirable template-free repair event and perhaps in any protocol aimed at creating a specific mutation, including base-editing and prime-editing. Base-editors consist of a nuclease-dead Cas9 fused to a mutagenic protein that will install mutations at certain positions in the protospacer targeted by the guideRNA^39,40^. HideRNAs could protect a specific desired mutation, provided they are delivered as in vitro assembled hideRNA:Cas9 RNPs. Yet another hideRNA application is the suppression of genome-wide Cas9 off-target activity, which can cause undesired gene disruptions with unexpected effects^5–7,16,34^ and indeed this application has recently been demonstrated^24,25^.

Although the rapidly developing gene editing field has created impressive feats of engineering that improved the recovery of single base-pair substitutions and decreased off-target DSB induction, ssODN-templated repair of Cas9-induced breaks remains a versatile and efficient method. The hideRNA approach we disclose here, makes use of ‘classic’ CRISPR-Cas9 components and can easily be implemented in the currently mostly-used and optimized protocols.

## Methods

### mESC GFP reporter cell line, tissue culture, transfection and flow cytometry

Mouse embryonic stem cells were cultured, transfected and analyzed by flow cytometry according to Harmsen et al.^26^. Briefly, IB10 mouse embryonic stem cells, maintained on MEF feeder layers in LIF-containing medium were transferred to 0.1% gelatin coated plates one day prior to transfection and cultured in Buffalo Rat Liver (BRL) cell conditioned medium. On the day of transfection, guideRNA or guide + hRNA expression vectors were transfected along with oligonucleotide repair templates with Transit-LT1 (Mirus) transfection reagent, according to manufacturer’s instructions. The day after transfection, cells were reseeded in BRL medium containing indicated amounts of puromycin and grown for 2 d, after which the medium was exchanged for medium without puromycin. After 2–3 d, cells were analyzed by flow cytometry using Cyan ADP flow cytometric analyzer (Beckman Coulter).

### Cas9, guideRNA and hRNA expression vectors

For plasmid-based delivery of Cas9 and guideRNAs we used ‘px330.PGKpur’, which is based on the popular px330 vector modified by us to contain a puromycin marker^26^. GuideRNAs were inserted as previously described^26^. Briefly; the vector was opened with the *Bbs*I or *Bpi*I restriction enzyme and the protospacer, a short dsDNA with 5′ overhangs on both sides (5′-CACC on the 5′ end and CAAA-5′ on the 3′ end) was introduced by ligation. To generate a vector expressing puromycin, Cas9, a guideRNA and a hideRNA, we first introduced the respective guideRNA protospacer. Then, we generated an additional cassette for hideRNA expression by first amplifying the empty guideRNA expression cassette from px330 using one perfectly matching primer [gRNA-rev (5′-AACGGGTACCTCTAGAGCC)] and one primer with a one-bp sequence alteration [gRNA-Fw (5′ CTTTTTACGGTACCTGGCCTTTTGC)] resulting in the U6-promoter + single-guideRNA containing fragment flanked by *Kpn*I sites. Then, we purify the resultant PCR product with the Qiaquick PCR purification kit (Qiagen) and digested it with KpnI-HF (New England Biolabs). This expression cassette was then ligated into *Kpn*I-linearized px330.PGKpur that already contained a guideRNA protospacer (in case of the experiments in Figs. 2, 3, 4, this was the spacer targeting GFP, GAAGCTCGATGCATAGGCCT, in case of the experiment in Fig. 5, these were the respective guides targeting the MMR genes). The resultant vector we refer to as the ‘gRNA + empty vector’. Finally, into this vector, the protospacer of the hideRNA was cloned, in the same way as the guideRNA protospacers were cloned to yield a vector expressing a guideRNA, hideRNA, Cas9 and puromycin.

### 293FT cell line, culture and transfection

293FT cells (Life Technologies) were cultured in optiMEM (Life technologies) with 8% FCS (Life Technologies). One day before transfection, 1.25E4 cells were seeded in a flat bottom 96-well. To transfect the 293FT cells, a mix of 50 ng gRNA or guideRNA + hRNA expressing vector and 150 ng ssODN HDR repair template DNA was prepared in 6.25 μl optiMEM medium (Life Technologies) in a 96-well plate, briefly mixed by pipetting. To this, 6.25 μl optiMEM containing 0.5 μl Transit-LT1 (Mirus) was added, and this was briefly mixed by pipetting. Complexes were formed for 15–30 min, and then transferred to 293FT cells of which the medium was gently refreshed 5–60 min before. Cells were then incubated for 16–20 h in 37 °C and 5% CO_2_, upon which the medium was refreshed with optiMEM containing 8% FCS and 7.2 μg/ml puromycin. Cells were incubated for 2 d, and then the medium was aspirated. Cells were washed with PBS (Life Technologies) and optiMEM with 8% FCS was added, followed by incubation for 2 d. Cells were detached by pipetting, and 1/10 of the cell suspension was transferred to a new flat bottom 96-well plate, and incubated for 2 or 4 d, after which the medium was aspirated and cell layers were frozen at − 20 °C.

### 293FT DNA isolation, PCR amplification, digestion and Caliper GX analysis

Genomic DNA was isolated from the frozen cell layers by addition of 50 μl of a solution containing 10 mM Tris pH 8.0, 100 mM NaCl, 0.5% SDS, 10 mM EDTA and 50 μl ml Proteinase K, followed by incubation for 2 h at 55 °C, and 45 min at 85 °C. Genomic DNA was precipitated by addition of 125 μl 100% Ethanol, followed by 30 min centrifugation at 4 °C at 4600 RCF. Precipitated DNA was washed with 70% Ethanol, air-dried and eluted overnight in 50 μl Tris/EDTA buffer (10 mM/0.5 mM).

The targets of gene modification were amplified by PCR with Taq polymerase (MRC Holland). Please refer to Supplementary Table 1 for the primer sequences and amplification conditions for each locus. For each locus and each sample, multiple PCR reactions were pooled and concentrated by precipitation (briefly: 0.1 volume of 3 M Sodium Acetate pH 5.0 and 0.7 volume of Isopropanol were added, followed by centrifugation at 10,000 RCF at 4 °C for 30 min, washing with 70% Ethanol, air-drying and DNA pellet elution in TE).

1 μl of purified PCR product was digested with 0.1 μl enzyme in a total volume of 2 μl in a PTC-100 thermocycler (MJ research) in PCR tubes for 3 h, otherwise according to manufacturer’s instructions (please refer to Supplementary Table 1 for the enzyme used for screening a particular modification). Each reaction was then supplemented with 18 μl of MQ H_2_O and briefly mixed, after which 15 μl of sample was analyzed on the Labchip GX (Caliper Life Sciences) to record the amounts of DNA at the cut and uncut positions.

### Zygote injections

Blastocyst injections were performed according to Pritchard et al.^41^ with some modifications. GuideRNAs and hRNAs were complexed with Cas9 protein (Alt-R, IDT) in separate reactions for 5 min at room temperature. 75 μl injection mix was prepared containing 1.875 μg guide RNA/15 μg Cas9 and 1.875 μg hRNA/15 μg Cas9 (if included) complex and 0.4 μM of HDR oligonucleotide. The injection mix was injected into the pronucleus of freshly isolated zygotes from the FVB/N strain. Zygotes were cultured in KSOM medium (Millipore) in a tissue culture dish at 5% CO_2_ and 37 °C until blastocyst state. Blastocyst were lysed in 5 μl of Direct Lysis tail buffer with 0.3125 mg/ml proteinase K for 30 min at 56 °C, followed by inactivation at 80 °C for 45 min. Then, the loci targeted by Cas9 nucleases were amplified using the entire blastocyst lysate as a template with Phusion flash High fidelity polymerase (NEB, F-548S), according to manufacturer’s instructions. Primer sequences are listed in Supplementary Table 1. A21V and R691W samples: and 1 μl of PCR product was sequenced using the primers shown in Supplementary Table 1. D304V samples: the entire volume of each blastocyst PCR was loaded on 2% agarose gel and different bands were separately isolated. Then, DNA was extracted from gel using QiaQuick gel purification kit, according to manufacturer’s instructions. The purified DNA was sequenced using the sequencing primer shown in Supplementary Table 1. None of the chromatograms deriving from a band migrating separately from the target site product could be algorithmically deconvoluted. The orientation of the sequencing primer was such that indels produced by re-cutting occurred between the sequencing primer and the HDR event in case of A21V, R691W and D304V.

Animal experiments were approved by the local Animal Ethical Committee at The Netherlands Cancer Institute and the National Commission for Animal Experimentation (Centrale Commissie Dierproeven) of The Netherlands and reported in accordance with ARRIVE guidelines.

### TIDER analysis

Algorithmic deconvolution of chromatograms using TIDER was performed using the Deskgen TIDER portal (http://TIDER.deskgen.com, accessed from July 30th 2019 through August 3^d^ 2019)^27^. The experimental HDR control samples (‘Reference chromatogram’) were constructed according to instructions^25^ using PCR products from control blastocysts and primers listed in Supplementary Table 1. Chromatograms from unmodified blastocysts were used as a wildtype control, in accordance with instructions accompanying TIDER. The TIDER result page was saved with firefox 68.0.2 (Mozilla Corporation) and the resultant HTML files containing the table with frequencies of the wildtype, various indels and the HDR sequence was transferred into an excel sheet using a custom VBA macro.

### Synthego ICE analysis

Synthego ICE analysis made use of the ICE build publically available on “https://github.com/synthego-open/ice” (version January 23, 2018, accessed June 3, 2019), according to the accompanying instructions. Around 30% of the chromatograms were of a quality that did not meet ICE standards and were excluded from analysis. Relevant ICE output was subsequently parsed into an Excel sheet using a custom Micrsoft Excel VBA macro in order to collect the fractions of wildtype, knock-in and indel alleles from each blastocyst. Only chromatograms with an R^2^ of 0.8 or higher (excluding around 1/3 of all analyzed chromatograms) were used for constructing Supplementary Fig. 4A.

All experiments were performed in accordance with Dutch and European legislation and guidelines.

## Supplementary Information

Supplementary Information 1.

Supplementary Information 2.

## Data Availability

The datasets generated during and/or analysed during the current study are available from the corresponding author on reasonable request.
