# Supplementary material for: HideRNAs protect against CRISPR-Cas9 re-cutting after successful single base-pair gene editing
Source: Sci Rep. 2022 Jun 10;12:9606. doi: 10.1038/s41598-022-13688-y (PMC9187658; doi:10.1038/s41598-022-13688-y)
Supplement: Supplementary file 1 — Supplementary Information 1. [file 41598_2022_13688_MOESM1_ESM.pdf]

Supplementary figure 1

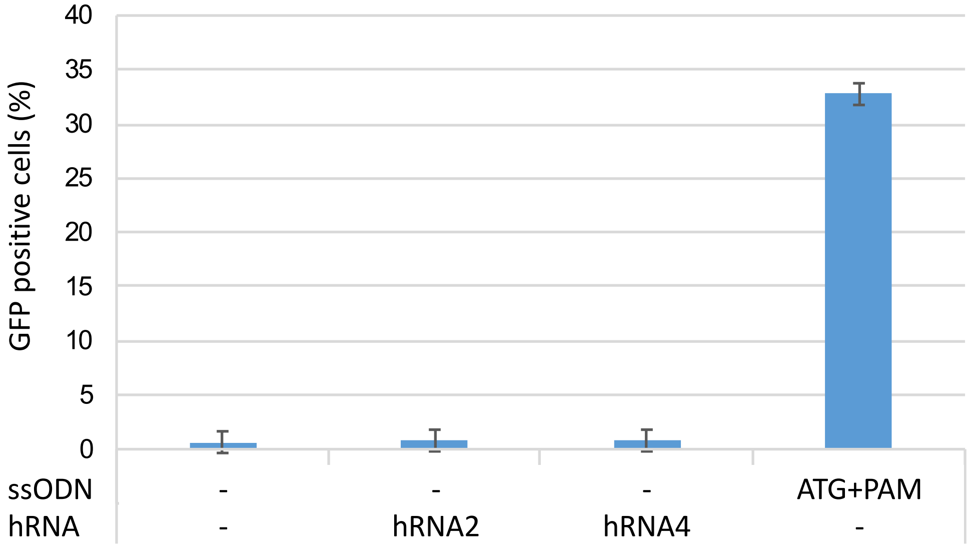

Supplementary figure 1. Mouse ES cells with the GFP reporter were transfected with vectors expressing puromycin, Cas9, a guideRNA or a guideRNA *plus* a 14 nucleotide hideRNA using either the same PAM as the guideRNA (hRNA2) or the 3 nucleotide distal PAM (hRNA4) without oligonucleotide (ssODN) template (-). As a positive control the ssODN ATG+PAM was used. Subsequently, cells were selected for puromycin and analyzed by flow cytometry for GFP expression.

Supplementary figure 2

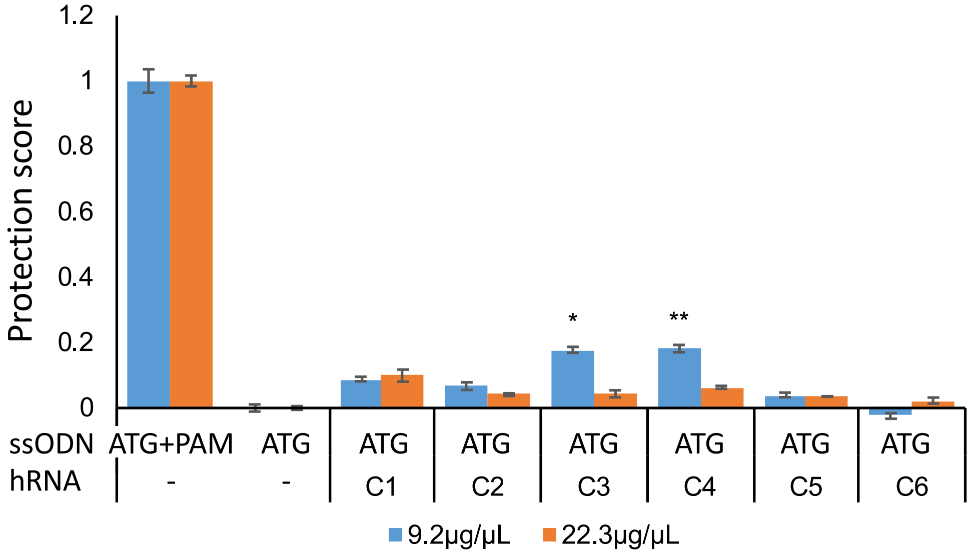

Supplementary figure 2. Protection score of six hideRNAs with unrelated spacer sequence, as in **Fig. 2**. The blue and red colors denote data from cells selected with 9.2 and 22.3 µg/mL puromycin, respectively. In all experiments, cells were transfected with the same guideRNA; ATG indicates the use of an ssODN only instructing the ATG-generating mutation; ATG+PAM indicates the use of an ssODN repair template instructing additional PAM disrupting base changes. C1 to C6 indicate hRNAs 1 to 6 with an unrelated spacer sequence.

Supplementary figure 3

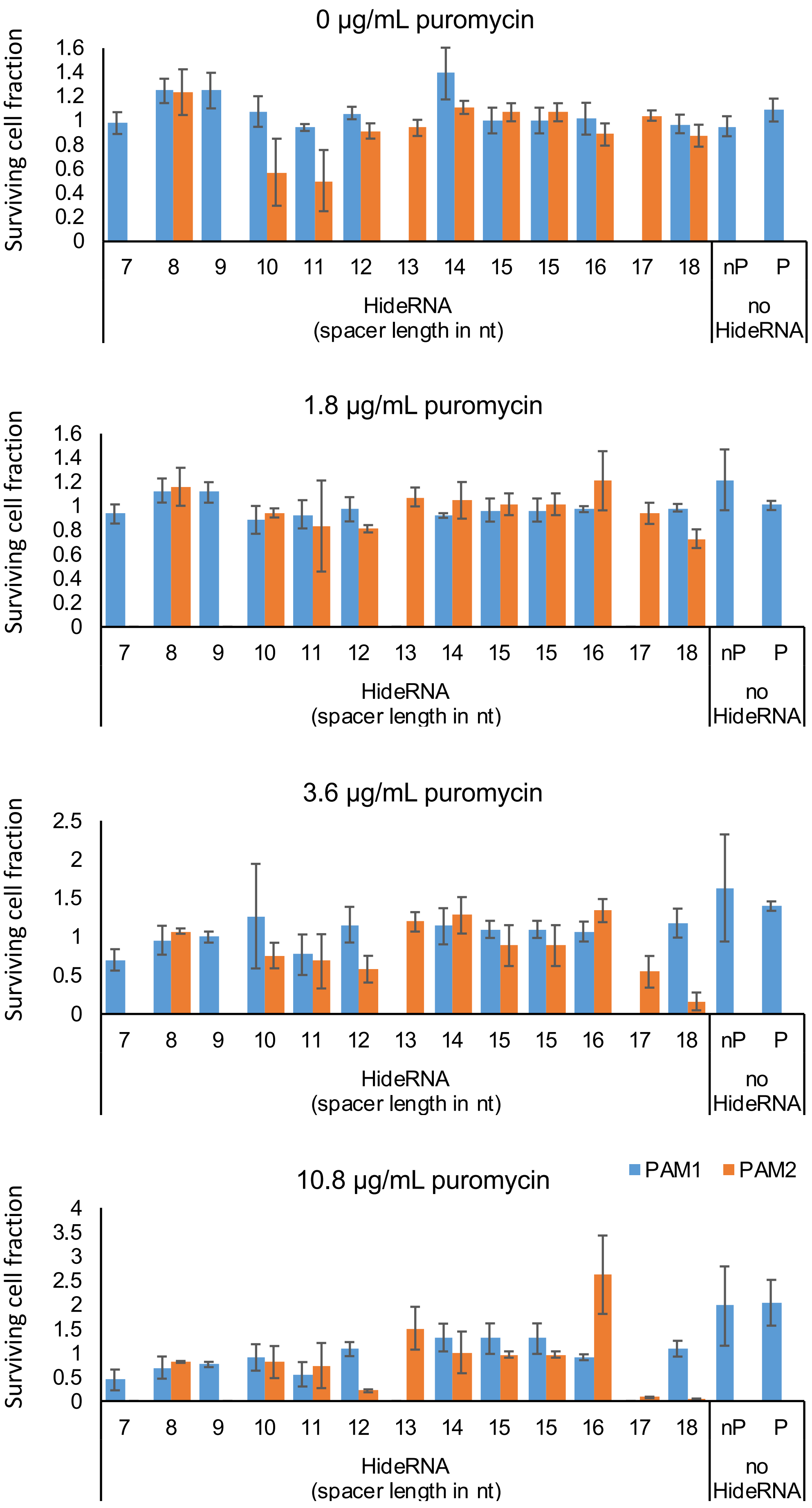

Supplementary figure 3. Live cell numbers in the hideRNA experiments from **Figure 3**, using hideRNA PAM 1 and PAM 2. The control conditions (guideRNA + ssODN, no hideRNA) are indicated as ‘no HideRNA’. In these conditions, data from the cells transfected with the ssODN that instructs a PAM disrupting mutation is designated with a ‘P’ while data from the transfections with ssODN that does not instruct a PAM mutation is designated with ‘NP’. For each puromycin concentration, the data was normalized. (n=3).

Supplementary figure 4

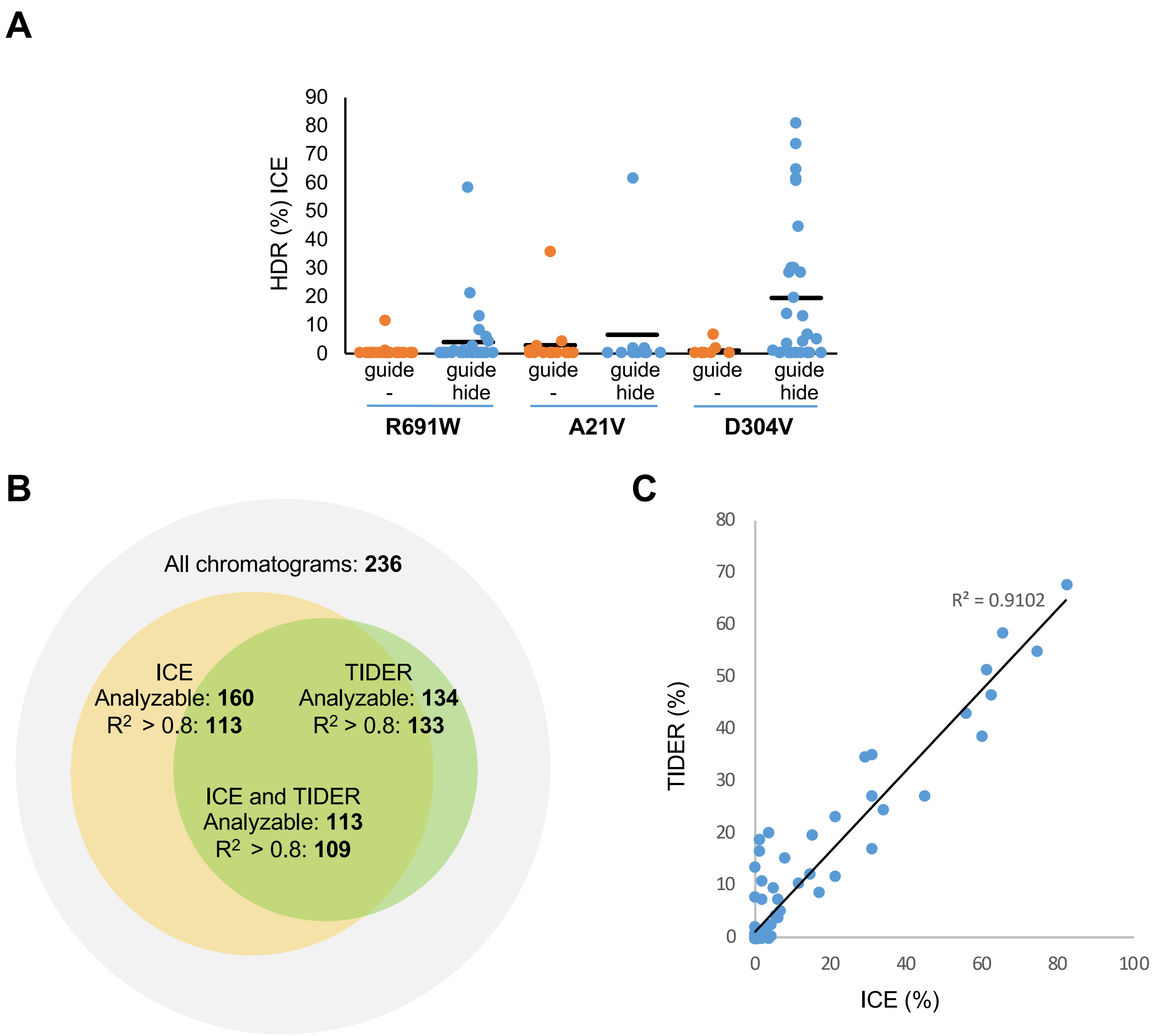

Supplementary figure 4. A. Fraction of HDR alleles per blastocyst, determined by Synthego ICE. The fraction of HDR for each individual blastocyst is represented as a dot. Averages for each condition are marked by the blacks lines. **B.** Summary of the analysis result of ICE and TIDER algorithms. ‘Analyzable’ means the number of chromatograms analyzed by the algorithm and pvalue >0.8 means the number of analyzed chromatograms with a R^2 >0.8 (over 80% of the sequences fitted to the chromatogram). **C.** Correlation of the HDR efficiency in chromatograms determined by ICE and TIDER with an R^2 exceeding 0.8.
